# Hospitals

**Published:** 1878-02

**Authors:** 


### Editorial.

HOSPITALS.—Three Christian virtues have been compared, but in the comparison charity is the greatest of all. "Corporations," it is said, "have no souls," but as the people have souls, and are, or should be, represented by municipal legislators, why is it that all cities of twenty thousand inhabitants do not build and support charitable institutions in which the helpless poor can be properly cared for when sick? The people of Atlanta exhibit evidences of this greatest virtue by devoting time, labor and money to private enterprizes for this purpose.

The Medical College clinic affords useful aid in this way, and whatever may be thought of the incentives, it is certainly true that the free surgical and medical treatment of the poor, as furnished in the Medical College clinic of this city, is one of the most useful charities among us. In making this statement we do not fail to recognize the praiseworthy efforts in this direction of those connected with the management of that charitable and useful institution, "The Ladies' Benevolent Home." The noble ladies engaged in this good work deserve, not only the thanks of helpless sufferers, but of the whole community, in which so many of this class are to be found. Not only so, but the blessings of Heaven will most assuredly attend those devoted to such Christian duties. No; we would not detract in the least from the importance of this institution founded in Christian charity, nor from the honor and praise of those engaged in this noble cause of humanity. To the extent of the donations they can obtain, added to their own contributions of money, labor and time, do these indefatigable ladies ameliorate the condition of this class of our people.

The charities bestowed at the Medical College are afforded entirely at the expense of money and labor by the College Faculty. In addition to those confined to bed, for whom bedding and fires are furnished, a larger number of out-door patients, afflicted with chronic diseases, are treated, than by the city physicians and all other charities put together. An average of over thirty cases are prescribed for weekly, and the medicine, surgical appliances, etc., furnished at the expense of the College Faculty. One hour each day, and, sometimes, two hours are devoted to this public service.

With shame citizens of Atlanta are compelled constantly to confess before the world that our city of thirty-five thousand inhabitants has never had a hospital, except such as have been inaugurated and supported by private enterprise, with the view of meeting the more pressing necessities of the poor. The city authorities still refuse to expend money for this noble object, notwithstanding the fact that it has been clearly shown that, by accepting the private aid offered, a hospital sufficiently commodious to fill the demand, can be built and supported, without larger expenditure of money than is now, almost uselessly, made on account of the sick poor. Salaries are paid to five city physicians. The amount not being sufficient, alone, to support them, they are necessarily allowed to do private practice to an unlimited extent. Now, until human nature is changed, or some special providence actuates physicians individually, paying patients will not be neglected in behalf of the other class. It is not because city physicians are worse than other people, but because of the defective plan adopted by the city authorities to take care of their pauper population.

